# Heart failure medication treatment and prognosis: a retrospective cross-sectional study

**DOI:** 10.3389/fphar.2025.1532123

**Published:** 2025-06-12

**Authors:** Qiankai Lin, Zongjie Lv, Daiyi Li, Qiao Ling, Sha Qiu, Xiaomei Lei, Fang Qin, Na Wang

**Affiliations:** ^1^ Department of Pharmacy, The Second Affiliated Hospital of Chongqing Medical University, Chongqing, China; ^2^ Department of Pharmacy, Chongqing Health Center for Women and Children/ Women and Children’s Hospital of Chongqing Medical University, Chongqing, China; ^3^ Department of Cardiology, The Second Affiliated Hospital of Chongqing Medical University, Chongqing, China

**Keywords:** heart failure, GDMT, retrospective cross-sectional study, logistic regression, readmission rate

## Abstract

**Objective:**

Heart failure (HF) is a significant global public health concern and the leading cause of morbidity and mortality worldwide, imposing a substantial economic burden on society. Guideline-directed medical therapy (GDMT) refers to the standardized pharmacological treatment for specific diseases based on recommendations from authoritative clinical guidelines and evidence from large-scale randomized clinical trials. GDMT serves as the cornerstone of drug therapy for heart failure patients. This study describes hospitalized HF patients and focuses on drug prescription and readmission rates.

**Methods:**

This study is a retrospective cross-sectional study with data from HF patients obtained from the Second Affiliated Hospital of Chongqing Medical University between January 2016 and June 2021. Patients were considered to have received GDMT if they were prescribed any guideline-recommended medication. Multilevel logistic regression was used to obtain the relationship between medication and readmission rates. The odds ratios (ORs) and 95% confidence intervals (CIs) have been reported.

**Results:**

In this study, a total of 5,356 HF patients (51.0% female; average age 77 years) were included. Among these patients, the most commonly used medications were mineralocorticoid receptor antagonists (MRA) (69.3%), Beta-blockers (54.2%), and lipid-lowering agents (46.0%). Currently, GDMT recommendations mainly include five types of drugs: diuretics, angiotensin receptor-neprilysin inhibitors (ARNIs), renin-angiotensin system inhibitors (ACEIs/ARBs), beta-blockers, mineralocorticoid receptor antagonists (MRAs), and sodium-glucose cotransporter-2 inhibitors (SGLT-2i). Among them, the utilization rates of ARNIs, SGLT-2i, triple therapy, and quadruple therapy are relatively low, accounting for 12.7%, 8.1%, 33.2%, and 3.75% respectively. The usage rates of these drugs are gradually increasing, especially after pharmacists participate in clinical decision-making and assist doctors in selecting therapeutic drugs, leading to a significant increase in the utilization rates of guideline-recommended drugs. Additionally, a multivariate logistic regression analysis of all drugs recommended by GDMT showed that ARBs (OR 0.681, CI 0.511–0.908), ARNIs (OR 0.191, CI 0.089–0.406), anticoagulants (OR 0.578, CI 0.403–0.829), tolvaptan (OR 0.340, CI 0.124–0.929), and SGLT-2i (OR 0.238, CI 0.058–0.969) significantly reduced the readmission rate of patients. Further subgroup analysis showed that the efficacy of the drugs varied slightly depending on the type of HF, but was consistent with guideline recommendations and clinical study results.

**Conclusion:**

In our hospital, the utilization rate of guideline-recommended drugs is gradually increasing, especially after pharmacists participate in rational drug use in clinical practice, the rate of increase is more significant, which is more in line with GDMT recommendations. Additionally, despite some limitations in our study, most of the guideline-recommended drugs show good therapeutic effects. And, we found that drugs such as SGLT-2i and ivabradine, despite their low usage rates, also demonstrate good therapeutic effects, providing significant implications for clinical decision-making.

## 1 Introduction

Heart failure (HF) refers to a condition of circulatory disorders caused by the inability of the heart to adequately pump venous blood due to impaired systolic and/or diastolic function, thereby resulting in venous congestion and insufficient arterial perfusion (Rogers and Bush, 2015). HF is characterized by symptoms such as shortness of breath, ankle swelling, and fatigue, alongside signs like elevated jugular venous pressure, pulmonary crackles, and peripheral edema (Metra and John R, 2017). As a rapidly progressing public health concern, it is estimated that there are currently over 64.3 million HF patients, and its prevalence continues to be on the rise (Savarese et al., 2023). The 30-day readmission rate of HF is as high as 20%–25% (Groenewegen et al., 2020). Furthermore, in 2012, the annual global healthcare costs for HF were over $108 billion and are expected to increase to $210 billion by 2030, thus creating a significant economic burden on society (Savarese et al., 2023). Extensive data also showed that each hospitalization decreases a patient’s health-related quality of life (HRQoL) and increases both 6-month and 1-year mortality rates (Setoguchi et al., 2007; Keidan et al., 2019).

Guideline-directed medical therapy (GDMT) refers to the standardized pharmacological treatment for specific diseases, particularly chronic conditions, based on recommendations from authoritative clinical guidelines and evidence from large-scale randomized clinical trials (Van der Meer et al., 2019). With objectives to improve survival rates, prevent recurrent hospitalizations, and enhance functional capacity, GDMT serves as the cornerstone of drug therapy for heart failure patients (Patel et al., 2023). According to “2016 ESC Guidelines for the diagnosis and treatment of acute and chronic heart failure” (Ponikowski et al., 2016) and “2018 Chinese Guidelines for the Diagnosis and Treatment of Heart Failure” (Liang, 2018), GDMT primarily includes five medication classes: diuretics, angiotensin receptor-neprilysin inhibitors (ARNI)/renin-angiotensin system inhibitors (ACEI/ARB), Beta-blockers, mineralocorticoid receptor antagonists (MRA), and sodium-glucose cotransporter-2 inhibitors (SGLT-2i). Therefore, one of the objectives of this study was to statistically analyze current medication usage among HF patients and identify existing treatment issues to promote GDMT implementation, ultimately improving patient outcomes and reducing disease burden.

In clinical practice, HF can be classified as heart failure with reduced ejection fraction (HFrEF, LVEF <40%), heart failure with mildly reduced ejection fraction (HFmrEF, 40% ≤LVEF <50%), and heart failure with preserved ejection fraction (HFpEF, LVEF ≥50%) (Lips and Cerny, 2022). Over time, pharmacological treatment for HF has evolved from a traditional approach focused on “positive inotropes, diuretics, and vasodilators” to the “triple-drug regimen” (ACEI/ARB + Beta-blockers + MRAs), and more recently, to novel HF medications, such as ARNIs and SGLT-2i. Currently, the pharmacological treatment for HFrEF primarily includes ACEIs, Beta-blockers, MRAs, and SGLT-2i, which have been shown to significantly improve patient prognosis (Rossignol et al., 2019). Previous studies have demonstrated that, compared to traditional therapies, a comprehensive approach combining multiple pharmacologic strategies can reduce cardiovascular hospitalization and mortality rates (Lim, 2020). Most medications that are effective for HFrEF do not demonstrate significant efficacy for HFpEF (Rossignol et al., 2019). Although some mediators of HFpEF are considered promising targets for pharmacological treatment, currently only the EMPEROR-Preserve randomized trial of empagliflozin has demonstrated the ability to reduce cardiovascular hospitalization and mortality rates in HFpEF patients (Packer et al., 2021). Additionally, there is a notable lack of prospective studies on HFmrEF. Thus, GDMT is critical for improving clinical outcomes in HF patients. Conversely, the absence of GDMT may lead to rapid disease progression and complications. Therefore, the other objectives of this study were to assess the relationship between different types of HF, the use of various medications, and readmission rates, thereby providing insights and evidence for clinical decision-making and promoting rational medication practices.

## 2 Materials and methods

### 2.1 Study design

This study is a retrospective cross-sectional study that aimed to assess the baseline characteristics of pharmacological treatment and readmission rates in HF patients. Clinical data were obtained from electronic medical records (EMRs) of HF patients of the Second Affiliated Hospital of Chongqing Medical University, encompassing patient demographics, clinical characteristics, laboratory and imaging results, medication prescriptions, and surgical records.

### 2.2 Study population

This study included patients diagnosed with HF at the Second Affiliated Hospital of Chongqing Medical University between January 2016 and June 2021, according to diagnostic criteria outlined in the “2016 ESC Guidelines for the diagnosis and treatment of acute and chronic heart failure “ (Ponikowski et al., 2016) and “2018 Chinese Guidelines for the Diagnosis and Treatment of Heart Failure” (Liang, 2018).

The patients enrolled in this study met the following criteria: (1) a discharge diagnosis of HF with the New York Heart Association (NYHA) functional classification II–IV; (2) complete medical records, with each hospitalization within 1 year considered an independent case; and (3) aged 18 years or older. Exclusion criteria included chronic systolic HF due to acute myocardial infarction, infective endocarditis, or acute myocarditis with a disease duration of less than 1 month; severe infections or malignancies; discharge or death within 1 day of admission; and incomplete medical records. The study protocol was reviewed and approved by the Ethics Committee of the Second Affiliated Hospital of Chongqing Medical University (Chongqing, China) (Approval No. 2023-7611).

### 2.3 Outcome measure

Patients were considered to have received GDMT if they were prescribed any guideline-recommended medication. The primary outcome of this study was the rate of readmission of patients within 30, 60, and 90 days after discharge.

### 2.4 Statistical analysis

Data were processed using SPSS version 26.0. To describe the data, we reported counts (n) and proportions (%). Normally distributed continuous variables (assessed using the Kolmogorov-Smirnov test) are presented as the mean ± standard deviation, with group comparisons for parametric data conducted using t-test or analysis of variance (ANOVA). Non-normally distributed continuous data are presented as the median with interquartile ranges (IQR), and group comparisons were performed using non-parametric rank-sum tests. Categorical data are expressed as a frequency and were analyzed using the chi-square test. The main variables included in the logistic regression analysis were various medications used for the treatment of HF. Odds ratios (ORs) and 95% confidence intervals (CIs) were reported. A p-value of <0.05 was considered statistically significant.

## 3 Results

### 3.1 Demographic characteristics of HF patients

**FIGURE 1:**
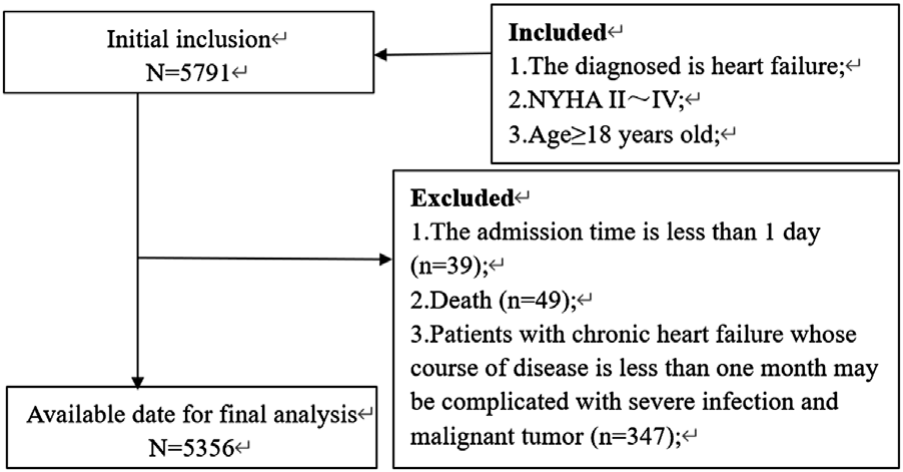
Study flowchart of HF patient selection.

An overview of the HF patient selection process is shown in Figure 1. From January 2016 to June 2021, a total of 11,377 patients were hospitalized for HF at our hospital. Of these, 5,356 patients were ultimately enrolled in the study. Table 1 presents the demographic characteristics of the enrolled patients. The patients had an average age of 77 ± 13 years, with 2,732 (51%) being female. Among those with a measured left ventricular ejection fraction (LVEF), the majority had LVEF values ≥50%, comprising 72.18% of the cohort. Additionally, patients with LVEF values of 40%–49% and <40% accounted for 11.50% and 16.32%, respectively. Patients with cardiac function graded as II, III, and IV accounted for 29.20%, 52.60%, and 18.10%, respectively. Furthermore, patients who had a history of smoking or alcohol consumption accounted for 25.60% and 15.20%, respectively, while hypertension (68.80%) and lung disease (64.80%) were the most common comorbidities among HF patients.

**TABLE 1:** Demographic characteristics of patients with HF.

Characteristics	$\bar{X}$ ±S/n (%)	Characteristics	$\bar{X}$ ±S/n (%)
Age, years	77 ± 13	High blood pressure	3685(68.80)
Female, n (%)	2732(51.0)	Coronary artery disease	1718(32.08)
Blood pressure, mmHg		Atrial fibrillation/flutter	2113(39.50)
Systolic pressure	133 ± 24	Diabetes	1869(34.90)
Diastolic pressure	78 ± 20	Lung disease	3471(64.80)
LVEF, n (%)		Occult coronary heart disease	12(0.20)
No measure	2930(54.70)	Dilated cardiomyopathy	436(8.10)
<40%	396(7.40)	Valvular heart disease	311(5.80)
40%–49%	279(5.20)	Stroke/TIA	71(1.30)
≥50%	1751(32.70)	Renal insufficiency	565(10.50)
NYHA, n (%)		COPD	997(18.60)
Ⅱ	1566(29.20)	Thyroid dysfunction	481(9.00)
Ⅲ	2818(52.60)	Ischemic	1510(28.20)
Ⅳ	972(18.10)	Dialysis/transplantation	33(0.60)
Smoke	1370(25.60)	Anemia	1180(22.00)
Drink	812(15.20)	Hyponatremia	138(2.60)

### 3.2 Overall medication usage in HF patients

#### 3.2.1 Monotherapy and combination therapy

**FIGURE 2:**
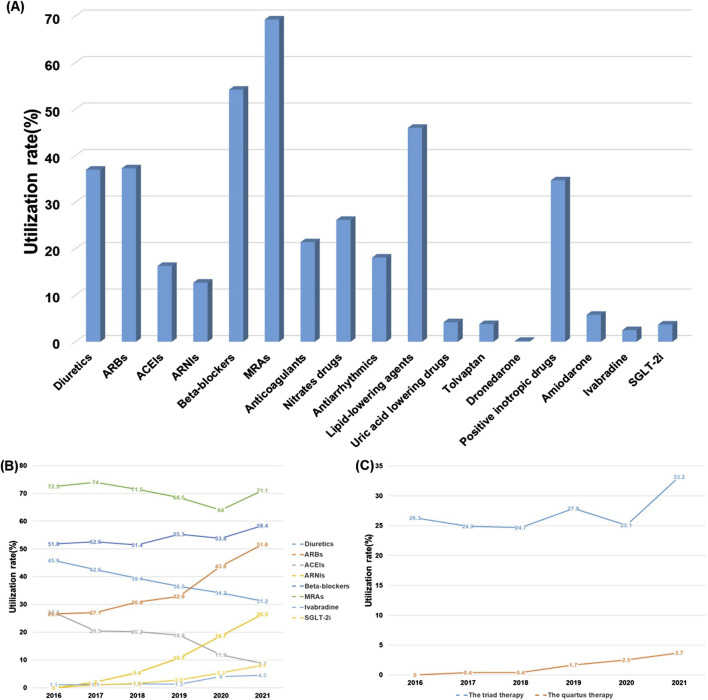
Overall medication usage in HF patients, January 2016-June 2021. **(A)** The overall use rate of each drug. **(B)** The change trend of the use rate of each drugs. **(C)** The change trend of the use rate of triple therapy and quadruple therapy

GDMT primarily included five medication classes. diuretics, ARNI/ACEI/ARB, Beta-blockers, MRA, and SGLT-2i. From January 2016 to June 2021, the medications with the highest overall usage rates were MRAs (69.3%), Beta-blockers (54.2%), and lipid-lowering agents (46.0%), while medications aimed at improving HF prognosis, such as ivabradine (2.5%) and SGLT-2i (3.7%), remained relatively underutilized (Figure 2A). With advancing understanding of heart failure and progress in clinical research, the guidelines informing GDMT have been continuously updated. In clinical practice, pharmacists collaborated with physicians to optimize medication selection based on the most current guideline recommendations, thereby enhancing medication quality and improving patient outcomes. During this period, the use of diuretics and ACEIs recommended for HF treatment by guidelines decreased annually (Lips and Cerny, 2022). In addition, there was a declining trend in the use of MRAs. Beta-blocker usage remained relatively stable. Moreover, the use of ARBs, ANRIs, SGLT-2i, and ivabradine exhibited an annual increase, though the usage rates of SGLT-2i and ivabradine remained low at only 8.1% and 4.5%, respectively (Figure 2B). For guideline-recommended ACEI/ARB/ARNI, Beta-blockers, and MRA triple therapy, and the SGLT-2i combined with ACEI/ARB/ARNI, Beta-blockers, and MRA quadruple therapy, usage rates increased annually but remained low at 33.2% and 3.7%, respectively. These trends demonstrated minimal fluctuations prior to 2018. Following the establishment of the heart failure center and subsequent GDMT implementation in 2018, with the assistance of pharmacists, the selection of treatment drugs became more reasonable and effective, and more in line with the recommendations of the guidelines (Figure 2C).

#### 3.2.2 Medication usage across different departments

**FIGURE 3:**
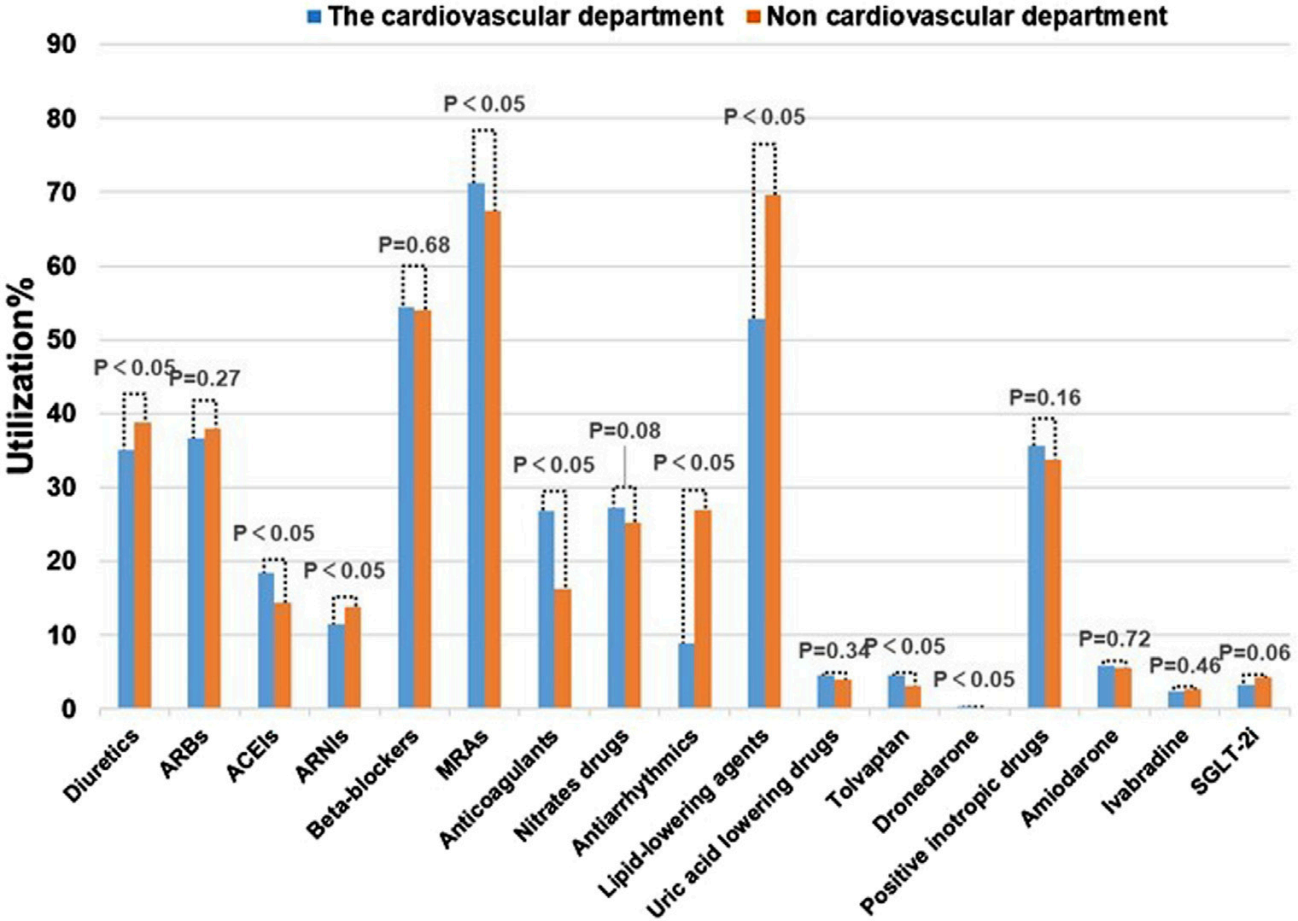
Comparison of drug therapy in patients with HF in different departments.

All patients were ultimately diagnosed with HF, however, they may have initially been admitted to different departments based on their initial symptoms. HF with various complications can also lead to differences in medication use. As shown in Figure 3, significant differences in the use of diuretics, ACEIs, ARNIs, MRAs, anticoagulants, antiarrhythmics, and other medications were observed across departments. For instance, in non-cardiovascular departments, diuretics were often utilized to manage edema resulting from liver or kidney disease, rather than alleviating HF symptoms. Over usage of antiarrhythmic drugs can also deviate from guideline recommendations. Patients in the cardiology department often present with comorbidities such as hypertension and atrial fibrillation, resulting in an increased usage of ACEIs, MRAs, and anticoagulants compared to other departments. These differences primarily stem from variations in initial symptoms and comorbidities. Furthermore, inappropriate medication practices in some of the departments may be an additional contributing factor. Therefore, clinical practice should promote rational medication use in accordance with GDMT and with input from pharmacists.

### 3.3 Comparison of medication usage in HF patients with different NYHA functional classifications

**FIGURE 4:**
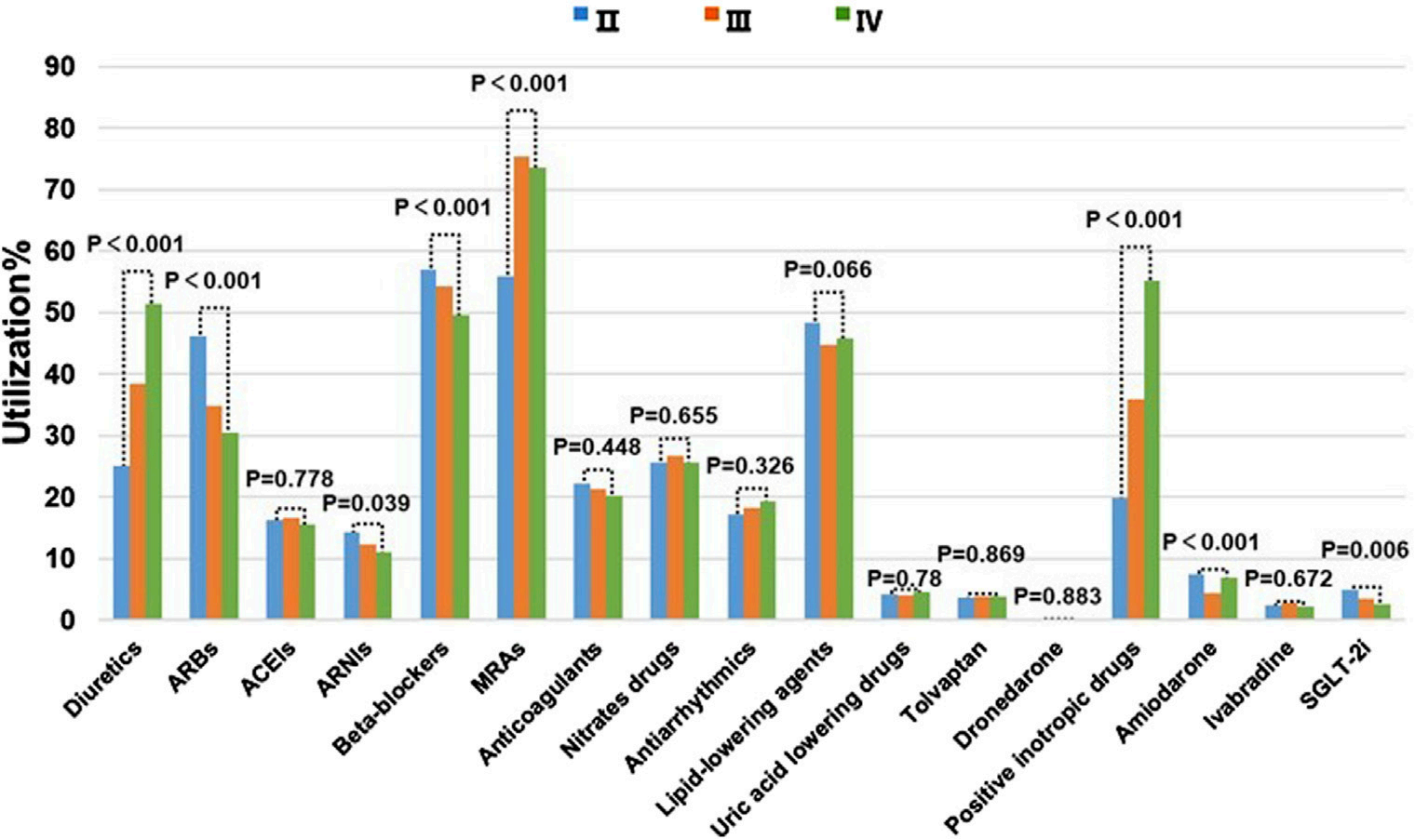
Comparison of Meditation usage in HF Patients with Different NYHA functional classifications.

According to a patient’s symptoms, heart function can be classified as class I to class IV to assess changes in symptoms during disease progression or with treatment. Medication usage also varies among patients with different NYHA functional classifications. Among the commonly used medications for HF, the usage rate of ACEIs does not show significant differences among the various NYHA functional classifications. Diuretics, MRAs, and positive inotropic drugs are more frequently prescribed for patients with more severe symptoms. In contrast, the usage rates of ARBs, Beta-blockers, and SGLT-2i are higher among patients with milder symptoms (Figure 4), which is consistent with the guideline recommendations.

### 3.4 Comparison of medication usage in patients with different types of HF

**FIGURE 5:**
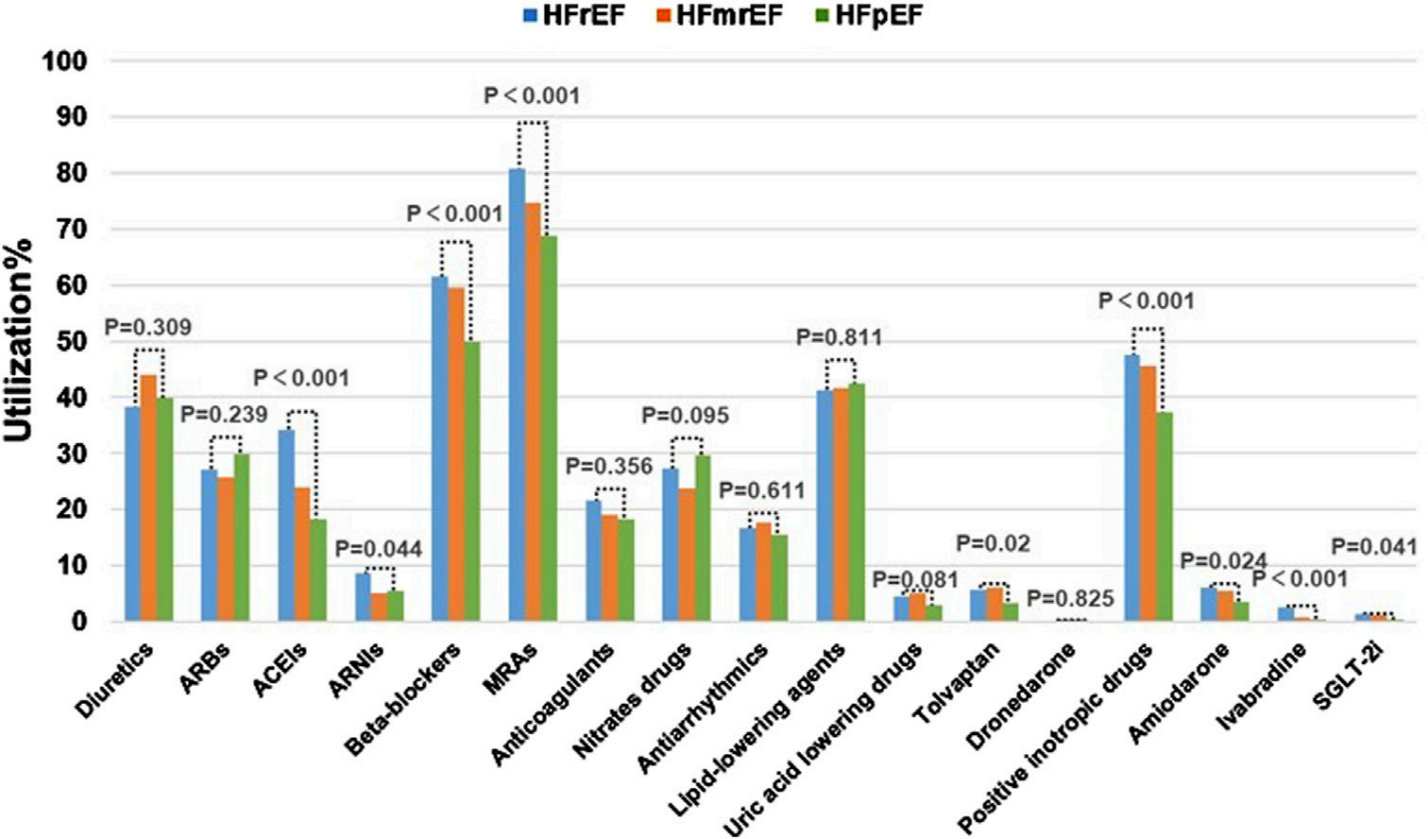
Comparison of Medication usage in HF Patients with Different LVEF values.

Based on the LVEF values, HF can be classified into three types: HFrEF, HFmrEF, and HFpEF. Of the 5,356 patients included in this study, LVEF values were not measured for some. Therefore, this section only focuses the 2,426 patients with recorded LVEF values. Among these patients, the usage rates of ACEIs, Beta-blockers, MRAs, and positive inotropic drugs showed significant differences across the different types of HF. The highest usage rates of MRAs (80.8%), Beta-blockers (61.6%), and positive inotropic drugs (47.5%) were observed in patients with HFrEF, and significantly exceeded the rates observed in patients with HFpEF and HFmrEF (Figure 5). These medications are exactly those that are recommended by guidelines for improving the prognosis of HFrEF patients.

### 3.5 Relationship between different NYHA functional classifications and types of HF and readmission rates

**FIGURE 6:**
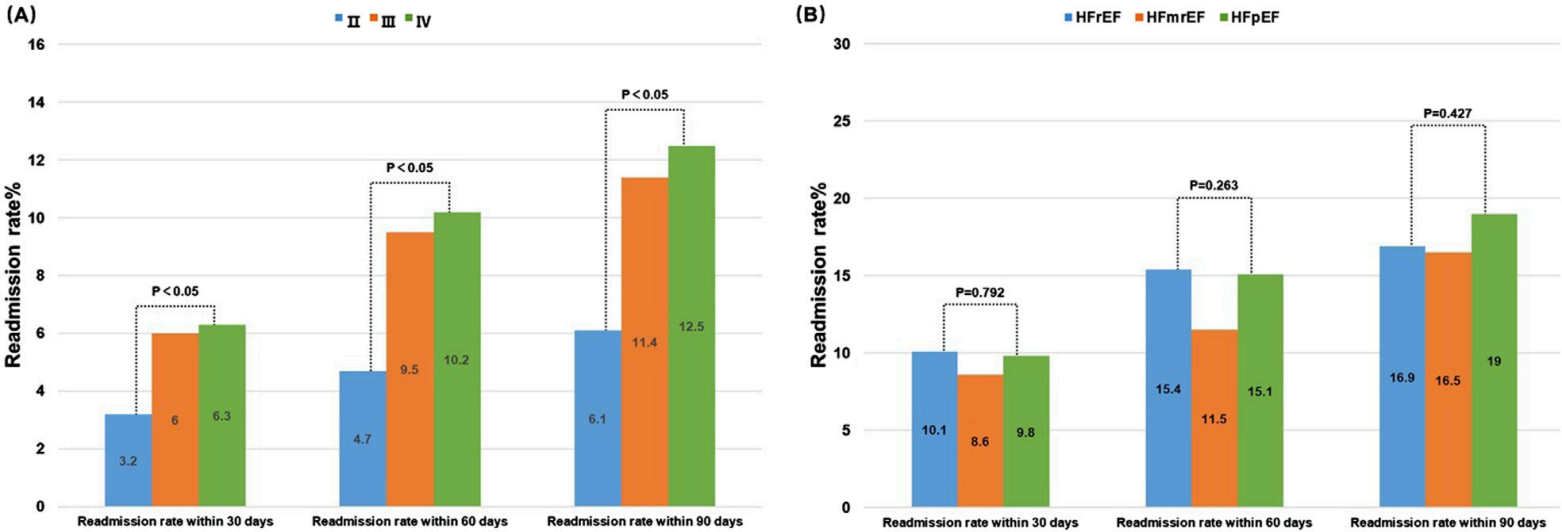
Relationship between readmission rates and different NYHA functional classifications **(A)** and different LVEF values **(B)**.

After effective in-hospital treatment and stabilization, the initial 3 months post-discharge are often referred to as the vulnerable period of HF. Therefore, the 3-month post-discharge readmission rate is an important indicator for assessing patient prognosis (Eltelbany et al., 2019). The NYHA functional classification reflects different levels of heart function severity, thereby suggesting a potential link between NYHA function classification and readmission rates. Statistical analysis of the 5,356 patients included in this study showed that patients classified as NYHA Class II had a significantly lower 3-month readmission rate compared to those classified as Class III and IV (p < 0.05). Although Class IV patients showed slightly higher readmission rates compared to those with Class III, this difference was not statistically significant. Among the different HF types, HFmrEF patients had the lowest 3-month readmission rate, but this difference was not statistically significant when compared with the other types. Additionally, regardless of NYHA class or LVEF in heart failure patients, the readmission rates within 30, 60, and 90 days progressively increased after discharge (Figure 6).

### 3.6 The impact of different medications on the readmission rate of overall HF patients

**FIGURE 7:**
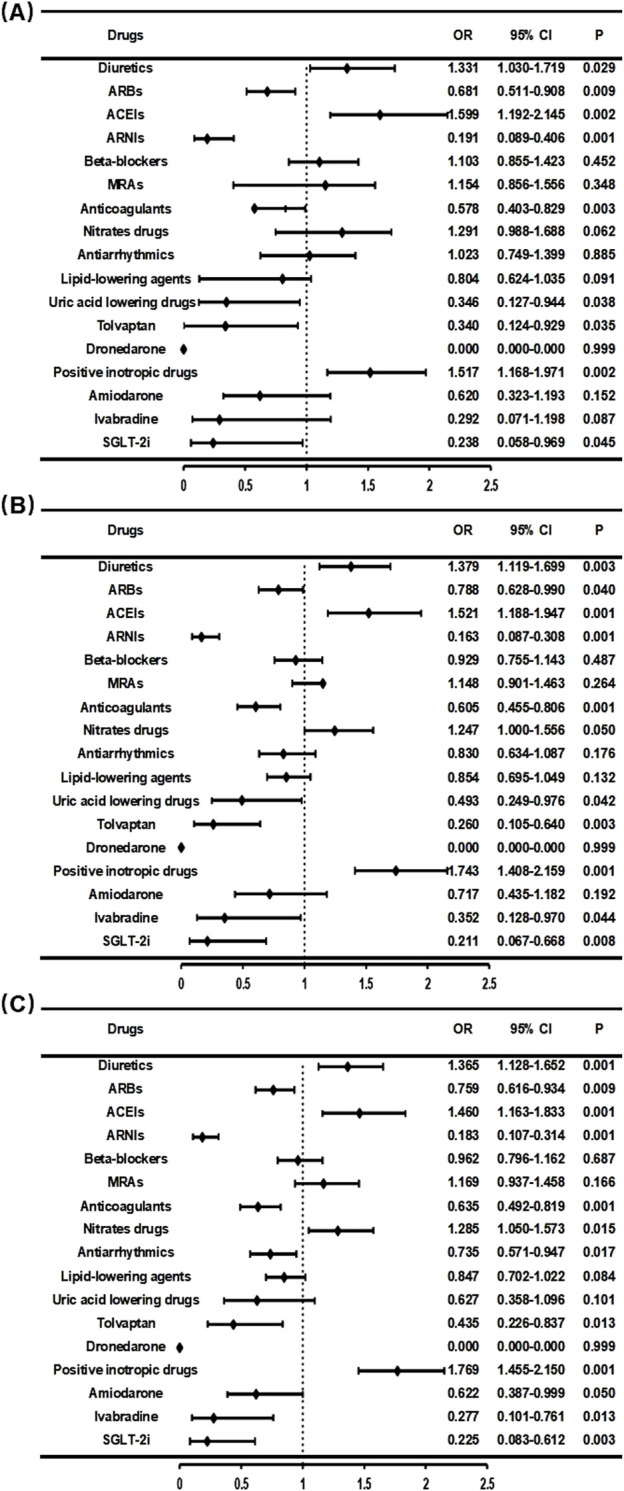
Relationship between drug therapy and readmission rate within 30 days **(A)**, 60 days **(B)**, and 90 days **(C)**.

In the previous part of this study investigated the impact of patient-specific factors, such as NYHA function classification and LVEF values, on the prognosis of HF. Furthermore, pharmacological interventions significantly influence patient outcomes, and GDMT can notably improve prognosis. Therefore, in this section, the effects of various medications on the prognosis of HF patients were examined. First, a comprehensive analysis of all patients included in this study was conducted, using readmission within 30, 60, and 90 days as outcome indicators. Next, logistic regression was performed to analyze the relationship between various medications and readmission rates, which revealed that ARBs (OR 0.681, CI 0.511–0.908), ANRIs (OR 0.191, CI 0.089–0.406), anticoagulants (OR 0.578, CI 0.403–0.829), uric acid lowering drugs (OR 0.346, CI 0.127–0.944), tolvaptan (OR 0.340, CI 0.124–0.929), and SGLT-2i (OR 0.238, CI 0.058–0.969) significantly reduced readmission rates within 30-day. The same impact was observed when analyzing the 60–90 days readmission rate of patients. Although ivabradine did not significantly affect readmission rates within 30 days, it substantially reduced readmission rates between 60 and 90 days (60-day: OR 0.352, CI 0.128-0.970; 90-day: OR 0.277, CI 0.101–0.761). In contrast, Beta-blockers, MRAs, nitrates drugs, antiarrhythmics, and lipid-lowering agents did not significantly impact readmission rates within 30–90 days (Figure 7).

### 3.7 Relationship between HFrEF patient medication usage and readmission rates

Several clinical studies have suggested that the impact of medication on the prognosis of patients with different types of HF may differ. Drugs that are effective in improving the prognosis of HFrEF do not necessarily have the same benefits on patients with HFpEF (Jhund et al., 2014; McMurray et al., 2014; Solomon et al., 2019). Consequently, administering the appropriate pharmacological treatment for the various types of HF can enhance patient prognosis. In this study, subgroup analysis of patients with different types of HF was performed to investigate the different effects of medications across various types of HF. For patients with HFrEF, GDMT includes the following medication classes: diuretics, renin-angiotensin system inhibitors (ACEIs, ARBs, or ARNIs), Beta-blockers, and MRAs (Liang, 2018). Logistic regression analysis was performed to examine the influence of various drugs on the prognosis of patients with HFrEF in this study, and showed that ANRIs (60-day: OR 0.118, CI 0.014-0.990; 90-day: OR 0.107, CI 0.013–0.887), MRAs (30-day: OR 0.286, CI 0.116–0.705), Anticoagulants (90-day: OR 0.404, CI 0.169–0.963), and Antiarrhythmics (90-day: OR 0.373, CI 0.140–0.993) ignificantly improved patient outcomes, while for other medications, no significant effect in treating HF was observed (Table 2).

**TABLE 2:** Relationship between medication usage and readmission rate in patients with HFrEF.

Drugs	Readmission rate within 30 days			Readmission rate within 60 days			Readmission rate within 90 days		
	OR	95% Cl	P	OR	95% Cl	P	OR	95% Cl	P
Diuretics	1.091	0.493–2.418	0.830	0.873	0.456–1.672	0.682	0.906	0.488–1.683	0.754
ARBs	0.528	0.195–1.427	0.208	0.952	0.464–1.954	0.893	1.031	0.520–2.045	0.930
ACEIs	2.316	1.058–50.72	0.036	1.762	0.916–3.389	0.090	1.739	0.926–3.268	0.085
ARNIs	0.000	0.000–0.000	0.998	0.118	0.014–0.990	0.049	0.107	0.013–0.887	0.038
Beta-blockers	1.928	0.844–4.406	0.119	1.713	0.879–3.335	0.114	1.503	0.799–2.830	0.206
MRAs	0.286	0.116–0.705	0.007	0.479	0.223–1.029	0.059	0.512	0.245–1.072	0.076
Anticoagulants	0.299	0.084–1.065	0.062	0.481	0.200–1.159	0.103	0.404	0.169–0.963	0.041
Nitrates drugs	0.928	0.407–2.118	0.859	0.766	0.384–1.530	0.451	0.716	0.367–1.397	0.328
Antiarrhythmics	0.497	0.163–1.513	0.218	0.425	0.159–1.139	0.089	0.373	0.140–0.993	0.048
Lipid-lowering agents	0.725	0.344–1.529	0.398	1.056	0.583–1.913	0.858	1.088	0.614–1.928	0.773
Uric acid lowering drugs	0.325	0.036–2.960	0.319	0.469	0.093–2.364	0.359	0.420	0.085–2.086	0.289
Tolvaptan	0.000	0.000–0.000	0.998	0.000	0.000–0.000	0.998	0.288	0.036–2.284	0.239
Dronedarone	0.000	0.000–0.000	0.999	0.000	0.000–0.000	0.998	0.000	0.000–0.000	0.000
Positive inotropic drugs	0.753	0.358–1.587	0.456	0.763	0.415–1.404	0.385	0.859	0.478–1.543	0.612
Amiodarone	1.866	0.368–9.465	0.451	0.807	0.172–3.780	0.786	0.715	0.153–3.330	0.669
Ivabradine	3.199	0.504–20.292	0.217	4.585	1.048–20.06	0.043	4.067	0.935–17.687	0.061
SGLT-2i	0.000	0.000–0.000	0.999	0.000	0.000–0.000	0.999	0.000	0.000–0.000	0.999

### 3.8 Relationship between HFmrEF patient medication usage and readmission rates

Currently, there is no large-scale prospective clinical study demonstrating the impact of medications on the prognosis of patients with HFmrEF. GDMT primarily consisted of diuretics, MRAs, and medications targeting underlying diseases and comorbidities (Liang, 2018). In this study, we performed logistic regression analysis to investigate the effects of various drugs on the prognosis of HFmrEF patients. The results showed that only Beta-blockers (30-day: OR 0.235, CI 0.087-0.635; 60-day: OR 0.342, CI 0.150–0.777) significantly reduced the readmission rate within 3 months, while other medications did not demonstrate any improvement in patient outcomes (Table 3).

**TABLE 3:** Relationship between medication usage and readmission rate in patients with HFmrEF.

Drugs	Readmission rate within 30 days			Readmission rate within 60 days			Readmission rate within 90 days		
	OR	95% Cl	P	OR	95% Cl	P	OR	95% Cl	P
Diuretics	0.925	0.372–2.302	0.867	1.437	0.649–3.186	0.371	2.273	1.133–4.560	0.021
ARBs	0.669	0.205–2.184	0.506	0.673	0.245–1.852	0.443	0.604	0.252–1.448	0.259
ACEIs	1.303	0.471–3.606	0.610	1.364	0.566–3.287	0.489	1.446	0.665–3.145	0.352
ARNIs	0.000	0.000–0.000	0.999	0.000	0.000–0.000	0.998	0.000	0.000–0.000	0.998
Beta-blockers	0.235	0.087–0.635	0.004	0.342	0.150–0.777	0.010	0.531	0.264–1.068	0.076
MRAs	1.485	0.472–4.676	0.499	1.533	0.553–4.254	0.412	1.508	0.614–3.706	0.370
Anticoagulants	0.348	0.074–1.626	0.180	0.265	0.059–1.190	0.083	0.428	0.139–1.318	0.139
Nitrates drugs	2.251	0.8246.153	0.114	1.802	0.740–4.387	0.195	1.644	0.746–3.621	0.217
Antiarrhythmics	0.825	0.241–2.822	0.759	0.588	0.182–1.903	0.376	0.350	0.119–1.035	0.058
Lipid-lowering agents	0.355	0.121–1.042	0.059	0.635	0.270–1.497	0.299	0.758	0.362–1.585	0.461
Uric acid lowering drugs	0.000	0.000–0.000	0.999	0.000	0.000–0.000	0.999	1.425	0.259–7.828	0.684
Tolvaptan	1.083	0.202–5.821	0.926	0.796	0.154–4.110	0.785	3.280	0.982–10.953	0.053
Dronedarone	0.000	0.000–0.000	0.999	0.000	0.000–0.000	0.999	0.000	0.000–0.000	0.000
Positive inotropic drugs	1.633	0.611–4.370	0.328	1.268	0.549–2.929	0.578	1.518	0.737–3.124	0.257
Amiodarone	0.000	0.000–0.000	0.998	1.542	0.288–8.251	0.613	0.879	0.174–4.437	0.876
Ivabradine	0.000	0.000–0.000	0.999	0.000	0.000–0.000	0.999	0.000	0.000–0.000	0.999
SGLT-2i	0.000	0.000–0.000	0.999	0.000	0.000–0.000	0.999	0.000	0.000–0.000	0.999

### 3.9 Relationship between HFpEF patient medication usage and readmission rates

Patients with HFpEF account for approximately half of all HF patients and HFpEF represents the type of HF with the highest morbidity and mortality (Komajda and Lam, 2014). Many clinical trials of medications targeting HFpEF patients have been conducted, and while these drugs have shown potential to improve patient prognosis, this has only been validated in the Phase III clinical trial of empagliflozin (Nassif et al., 2021). Clinical trials have failed to demonstrate that ACEI/ARBs or beta-blockers improve clinical outcomes or reduce mortality in HFpEF patients. Due to the marked heterogeneity in the pathophysiological mechanisms of HFpEF, current GDMT guidelines recommend only diuretics and MRAs for heart failure management in this population, while emphasizing treatment of underlying comorbidities and primary diseases (Liang, 2018).In this study, it was demonstrated that ANRI significantly reduced the readmission rate of HFpEF patients within 60–90 days (60-day: OR 0.232, CI 0.084-0.640; 90-day: OR 0.269, CI 0.116–0.624) (Table 4). These findings offer new evidence for guiding the selection of clinical medication. Subgroup analysis across the three types of HF was performed and showed that, although ANRI, ivabradine, and SGLT-2i were used infrequently, the common observation was that all showed favorable effects across different HF patient groups with almost no readmissions within 90 days among those taking these medications. While the sample size was small and lacks statistical significance, these findings still offer meaningful reference points for clinical decision-making in medication selection.

**TABLE 4:** Relationship between drug use and readmission rate in patients with HFpEF.

Drugs	Readmission rate within 30 days			Readmission rate within 60 days			Readmission rate within 90 days		
	OR	95% Cl	P	OR	95% Cl	P	OR	95% Cl	P
Diuretics	1.332	0.951–1.867	0.095	1.270	0.959–1.683	0.095	1.211	0.936–1.567	0.146
ARBs	0.807	0.557–1.171	0.260	0.917	0.676–1.243	0.576	0.850	0.642–1.125	0.257
ACEIs	0.998	0.653–1.525	0.991	0.992	0.695–1.415	0.963	0.951	0.687–1.317	0.763
ARNIs	0.381	0.138–1.056	0.063	0.232	0.084–0.640	0.005	0.269	0.116–0.624	0.002
Beta-blockers	1.241	0.889–1.733	0.204	1.036	0.785–1.367	0.803	1.115	0.865–1.437	0.399
MRAs	1.271	0.862–1.875	0.227	1.194	0.868–1.643	0.275	1.240	0.927–1.660	0.147
Anticoagulants	0.827	0.534–1.282	0.396	0.708	0.486–1.030	0.071	0.799	0.574–1.112	0.183
Nitrates drugs	1.078	0.758–1.535	0.675	1.043	0.776–1.402	0.781	1.118	0.854–1.464	0.415
Antiarrhythmics	1.324	0.875–2.003	0.184	1.100	0.766–1.578	0.606	1.009	0.720–1.414	0.958
Lipid-lowering agents	0.900	0.645–1.254	0.532	0.930	0.706–1.226	0.607	0.920	0.715–1.184	0.517
Uric acid lowering drugs	0.186	0.025–1.365	0.098	0.468	0.165–1.327	0.153	0.675	0.297–1.538	0.350
Tolvaptan	0.309	0.074–1.296	0.108	0.283	0.087–0.925	0.037	0.217	0.067–0.705	0.011
Dronedarone	0.000	0.000–0.000	0.999	0.000	0.000–0.000	0.999	0.000	0.000–0.000	0.999
Positive inotropic drugs	1.331	0.946–1.873	0.101	1.681	1.264–2.235	0.000	1.667	1.284–2.163	0.000
Amiodarone	0.937	0.391–2.245	0.883	1.031	0.507–2.094	0.934	0.832	0.421–1.643	0.597
Ivabradine	0.000	0.000–0.000	0.999	0.000	0.000–0.000	0.999	0.000	0.000–0.000	0.999
SGLT-2i	0.000	0.000–0.000	0.999	0.000	0.000–0.000	0.999	0.000	0.000–0.000	0.999

## 4 Discussion

With the current advances in scientific research, HF management has evolved from the initial approaches of cardiotonic agents, diuretics and vasodilators, the triple therapy (ACEI/ARB + Beta-blockers + MRA) and now to the quadruple therapy (ACEI/ARB/ARNI + Beta-blockers + MRA + SGLT-2i), thus continuously improving the prognosis of HF (JoAnn et al., 2010; McMurray, 2013; Lips and Cerny, 2022). Moreover, recent data has indicated that the novel quadruple therapy has significant benefits on HF outcomes (Gwag et al., 2018; Lund et al., 2018). The 2014 PARADIGM-HF study showed that, compared to standard treatment (Rogers and Bush, 2015), replacing ACEI with sacubitril/valsartan in HFrEF patients lowered the risk of cardiovascular death and HF hospitalization by 20% and reduced all-cause mortality by 16%. Consequently, ARNI is now preferred over ACEI/ARB in HFrEF pharmacotherapy (Ostios Garcia, 2015). The data obtained in the current study has shown that ANRI has gradually replaced ACEI in HF treatment. However, in the current study, guideline-recommended Beta-blockers did not significantly improve HFrEF prognosis, which was likely due to dosages not reaching the recommended treatment levels. In addition, in some studies, it was indicated that ACEI/ARB, Beta-blockers, and MRA medications may benefit HFmrEF patients by potentially improving their prognosis. HFmrEF patients frequently have comorbid hypertension and coronary artery disease, making ACEI/ARB and Beta-blockers therapies appropriate for managing these conditions (Cleland et al., 2018). The recently completed EMPEROR-Preserved study reported that empagliflozin, compared with the placebo, reduced the risk of cardiovascular death or HF hospitalization by 21% and significantly, early, and consistently lowered the risk and severity of both hospitalization and outpatient HF events in HFpEF patients (Nassif et al., 2021). In our study, patients who received SGLT-2i therapy also demonstrated improved prognosis.

Pharmacists play a significant role in GDMT. They assist doctors in selecting appropriate medications, adjusting dosages, and evaluating drug interactions based on the recommendations of the latest guidelines, while also considering the characteristics of individual patients. Additionally, when contraindications exist, they recommend suitable alternative medications as per the guidelines. Guidance on medication use and lifestyle habits for patients also enhances treatment adherence. This approach enhances treatment quality and ensures safety and effectiveness. In addition, variations in medication usage are influenced by multiple factors, including regional healthcare levels, awareness of HF and its treatment regimen, patient adherence to treatment, and economic conditions. As mentioned earlier, there are also notable differences in HF medication use across departments within our hospital. The data obtained in our study also revealed that guideline-recommended medication usage in our hospital was lower than the national average, with particularly low use rates of triple and new quadruple therapies. Compared to the U.S. GWTG-HF study (Figueroa et al., 2020), the MRA usage from patients in our hospital was higher, but ACEI/ARB/ARNI and BB use rates were lower. This may be because MRA drugs are also frequently used to improve short-term HF severity and, in our setting, may act as diuretics. In recent years, the utilization rate of guideline-recommended medications has been increasing annually, with a significant rise after 2018. This trend may be attributed to the establishment of heart failure centers and the participation of pharmacists in clinical decision-making, which have promoted the implementation of GDMT and are of great significance for improving HF outcomes.

In this study, the 30-day readmission rate for HF patients discharged from our hospital was 5.2%, and increased to 10.1% within 90 days. This increase may relate to suboptimal dosing or inconsistent medication use, which is especially the case for newer HF drugs such as ARNIs and SGLT-2i, which are underutilized. The results obtained from analyzing the relationship between various medications and readmission rates do not fully align with previously reported study findings (Bozkurt, 2024). For example, diuretics, ACEIs, and positive inotropic drugs not only failed to improve patient prognosis but were also associated with higher readmission rates, thus diverging from guideline recommendations and clinical expectations. This discrepancy may result from the retrospective nature of the current study, in which only data from HF patients in our hospital between January 2016 and June 2021 were collected. The data used for this study may have missing information and potential bias, as well as a single-center approach in which only readmissions to our hospital were tracked, and data on patients readmitted elsewhere were not taken into account-our study’s largest limitation. Nonetheless, most findings align with data presented in the existing literature. Based on our findings, newer anti-HF drugs such as ARNIs and SGLT-2i, along with triple and quadruple therapy regimens were shown to effectively improve patient outcomes, thereby offering greater options and evidence for optimizing clinical treatment of HF. Early, low-dose use of new quadruple therapy (ARNI/ACEI/ARB + Beta-blockers + MRA + SGLT-2i), with gradual titration to target or maximally tolerated doses and long-term maintenance, may provide an effective strategy for significantly reducing readmission and mortality rates and improving HF prognosis (Rossignol et al., 2019).

## 5 Strengths and limitations

The strength of this study lies in its large sample size (*n* = 5356), including accurate, reliable data that covers a diverse population, thus ensuring a degree of representativeness. However, as a retrospective study, this study is affected by missing data and potential bias and therefore, there is a certain time lag in the findings. Additionally, the single-center date is another limitation of this study. Nevertheless, this study assesses the real-world impact of various drugs on HF prognosis, thereby providing significant guidance for clinical drug selection. Future plans involve a large-scale, multicenter, prospective clinical study to validate the findings and to more accurately assess the impact of different drugs on HF prognosis.

## 6 Conclusion

Cardiovascular disease has become the leading cause of death in China, with the incidence of HF increasing rapidly. The mortality rate of HF, comparable to that of cancer, makes it a significant public health concern both domestically and globally. Currently, the utilization rate of guideline-recommended medications in our hospital is significantly lower than the national and international averages. In recent years, this situation has improved, especially since 2018 when pharmacists began participating in clinical decision-making, assisting doctors in rational drug use. As a result, the utilization rate of GDMT drugs has increased significantly compared to previous years. Additionally, we evaluated the impact of all medications on the prognosis of patients with HF. Despite some limitations in our study, most guideline-recommended medications demonstrated good therapeutic effects during the treatment process. Moreover, novel drugs for HF treatment, such as SGLT-2i and ivabradine, despite their low usage rates, still exhibited favorable efficacy. This provides additional evidence for rational drug use in clinical practice, contributing to improved patient outcomes.

## Data Availability

The original contributions presented in the study are included in the article/supplementary material, further inquiries can be directed to the corresponding authors.
